# Symptomatic presentation influences outcomes in pediatric restrictive cardiomyopathy

**DOI:** 10.3389/fped.2023.1264751

**Published:** 2023-10-19

**Authors:** Melissa Lorenzo, Aine Lynch, Jenna Ashkanase, Linda Fazari, Kristen George, Katelyn Arathoon, Sunghoon Minn, Dawn Nicolson, Aamir Jeewa, Emilie Jean-St-Michel

**Affiliations:** ^1^ Department of Pediatrics, The Hospital for Sick Children, University of Toronto, Toronto, ON, Canada; ^2^ Department of Pediatrics, McMaster Children's Hospital, McMaster University, Hamilton, ON, Canada

**Keywords:** restrictive cardiomyopathy, symptoms, outcomes, heart transplant, transplant-free survival

## Abstract

**Introduction:**

Children with restrictive cardiomyopathy (RCM) traditionally have a poor prognosis, with most patients either dying or requiring heart transplantation within 2 years of diagnosis. The development of symptoms in RCM suggests advanced disease. However, as screening practices evolve and lead to diagnosis of early disease, identifying appropriate timing of transplant listing becomes increasingly important. In this context we compared outcomes of children with RCM presenting with clinical symptoms to those asymptomatic at initial presentation.

**Methods:**

This retrospective cohort study included 25 patients with RCM presenting to a quaternary care center between 2001 and 2018. Times to transplantation, death, and a composite outcome of adverse cardiac events (CPR, cardioversion, inotropic support, mechanical ventilation, mechanical support, or heart transplant) were compared between those symptomatic and asymptomatic at presentation.

**Results:**

At 2 years following diagnosis, patients asymptomatic at presentation had a significantly better transplant-free survival at 57% compared to 17% for symptomatic patients (*p* = 0.03). Those asymptomatic at diagnosis also had significantly improved cardiac event-free survival at 71% compared to symptomatic patients at 25% (*p* = 0.01). In multivariable analysis, cardiac symptoms at presentation remained an independent risk factor for heart-transplant or death [hazard ratio 5.17 (1.28–20.85), *p* = 0.02].

**Conclusion:**

Patients with RCM who are symptomatic at time of diagnosis have significantly worse transplant-free survival and cardiac event-free survival. Given current practice variability in timing of transplant listing, the presence of any cardiac symptoms is an important negative prognostic marker and should prompt urgent transplant listing.

## 1. Introduction

Restrictive cardiomyopathy (RCM) is the least common form of cardiomyopathy, accounting for 2%–5% of pediatric cardiomyopathies (1–3). RCM is characterized by diastolic dysfunction, with normal or decreased volume of both ventricles associated with biatrial enlargement, normal left ventricular wall thickness and atrioventicular valves, and impaired ventricular filling with restrictive physiology (3, 4).

Pediatric RCM has been associated with worse outcomes than other forms of cardiomyopathies with approximately 50% of patients either dying or requiring heart transplant within 2 years of diagnosis (2, 5–12). This is contributed to at least in part by challenges in establishing diagnosis due to absence of symptoms in early disease and difficulties in using currently available mechanical circulatory support (MCS) options to support RCM patients in end-stage heart failure to transplant (13). Of those who do present with symptoms, presentation varies from palpitations, chest pain, syncope, congestive heart failure (CHF) symptoms, and even sudden cardiac death, which has been attributed to subendocardial ischemia secondary to high diastolic pressures (1, 3, 14). Unlike cardiomyopathies characterized by systolic ventricular dysfunction, where evidence-based guidelines exist on timing of heart transplant listing based on clinical status and heart failure severity, risk factors for death in RCM are less well-established and listing practices are largely center dependent (2, 15–17).

As cardiomyopathy screening practices evolve, and children are identified at earlier disease stages, ascertaining those patients in whom there is a clear survival benefit from early listing for transplant becomes critical. Therefore, our study sought to ascertain transplant-free survival and cardiac event-free survival in pediatric RCM patients who present with and without cardiac symptoms at time of diagnosis.

## 2. Materials and methods

### 2.1. Construction of the study cohort

The SickKids Heart Failure (HF) database is a retrospective cohort study of pediatric patients with cardiomyopathy or HF seen at a single, quaternary cardiac transplant center, the Hospital for Sick Children, Toronto, ON and diagnosed between January 2001 and July 2018 (18). Subjects were identified by screening the electronic medical record and the electronic echocardiogram (echo) database for the diagnosis of “restrictive cardiomyopathy”. Subjects were considered to meet diagnostic criteria for RCM if they had a documented diagnosis by a HF cardiologist and demonstrated evidence on echo of normal or reduced ventricular volumes, with atrial enlargement, normal ventricular wall thickness and atrioventricular valves, and diastolic dysfunction (4). Patients with increased posterior wall thickness and/or septal thickness (*z*-score >2) were reviewed by 2 HF pediatric cardiologists (EJ and AJ). Other subtypes that were identified were those with RCM and significant ventricular hypertrophy (RCM with HCM features), but who did not meet criteria for HCM (19). Similarly, patients with increased myocardial trabeculation, but who did not fulfill diagnostic criteria for left ventricular non compaction (LVNC), were reviewed and included if the patients were felt to have predominantly RCM; these patients were classified as RCM with features of left ventricular non compaction (RCM with LVNC features) (20). Therefore, these 2 groups of patients were included as they would not have fit into a primary diagnosis of either HCM or LVNC.

Subjects were excluded if they were 18 years of age or older, had congenital heart disease, metabolic disease, or were never evaluated by a cardiologist at our institution. The RCM patients were then divided into those without cardiac symptoms (asymptomatic patients) to patients with cardiac-related symptoms (symptomatic patients) at the time of initial diagnosis.

### 2.2. Data collection

Baseline characteristics including sex, age at diagnosis, status at diagnosis, family history of cardiomyopathy, and pathogenic genetic mutation were compared between the asymptomatic and symptomatic RCM patients. Cardiac investigations including basic functional echos, electrocardiograms (ECGs), cardiac magnetic resonance imaging (MRI), and Holter monitors at diagnosis and during follow-up were also compared between the 2 groups. Echo *z*-scores were calculated relative to body-surface area as per the Pediatric Heart Network calculator (21). The presence of ectopy on ECG was defined as frequent premature ventricular contractions or non-sustained ventricular tachycardia (VT) (3 or more beats at >120 beats per minute). On Holter monitoring, the presence of ectopy was defined as >500 ventricular extrasystoles in 24 hours, runs of ventricular ectopy of >30 seconds in duration, or non-sustained VT (3 or more beats at >120 beats per minute for < 30 seconds). Ventricular tachycardia was defined as sustained ventricular arrhythmia at >120 beats per minute for >30 seconds (19, 22). Signs of ischemia were defined as episodic T wave inversion, new ST depression with cardiac symptoms, or ST depression >3 mm on Holter monitoring.

Cardiac care needs during follow-up were also compared, including use of heart failure medications, number of cardiac outpatient visits, cardiac hospitalizations, and intensive care unit (ICU) admissions.

### 2.3. Endpoints

Study participants were followed from date of diagnosis until end of the study period, or until death, heart transplant, or transition to adult cardiac care. Primary outcome was transplant-free survival. Secondary outcomes included cardiac event-free survival, defined as survival without incurring cardiopulmonary resuscitation (CPR), cardioversion, inotropic support, mechanical ventilation, MCS, or heart transplant. Time to symptom onset was examined for patients who were asymptomatic at diagnosis.

### 2.4. Statistical analysis

Descriptive statistics were reported as mean ± standard deviation or median and interquartile range (IQR). Baseline, operative, and echo characteristics were compared between the asymptomatic and symptomatic patients at time of RCM diagnosis using Chi-square, student's *t*-test, and Wilcoxon rank-sum tests as appropriate. Simple group comparisons were made with the log-rank test and displayed using the Kaplan–Meier method.

Candidate variables were identified for risk factor analysis. Bootstrap aggregation with resampling (500 resamples) was used for variable selection (23). Candidate variables that were found in at least 25% of the bootstrap models were subjected to a backward selection, with a retention threshold of *p* < 0.05. The final multivariable Cox regression model was obtained. The level of statistical significance was set at *p* = 0.05. Statistical analyses were performed using SAS 9.4 software Statistical Analysis System (RRID:SCR_008567).

## 3. Results

### 3.1. Baseline characteristics of cohort

A total of 25 patients with RCM met inclusion criteria, of whom 7 (28%) were asymptomatic at diagnosis and 18 (72%) were symptomatic (Table 1). Most symptomatic patients presented with CHF 14 (78%), with the remainder presenting with cardiac arrest 2 (11%), chest pain 1 (4%), or hemoptysis 1 (4%). Asymptomatic patients were younger at diagnosis (age <3 years: 100% vs. 39%, *p* = 0.05) and were more likely to be outpatient at time of diagnosis (86% vs. 28%, *p* = 0.05). There were no significant differences between groups for presence of a pathogenic mutation in MYH7, MYBPC3, or TTN (29% vs. 39%, *p* = 1.00), or a first degree relative with cardiomyopathy (14% vs. 11%, *p* = 0.83) (Table 1).

**Table 1:** Baseline characteristics and investigations at the time of diagnosis of RCM.

	Total RCM Cohort *n* = 25	Asymptomatic *n* = 7 (28%)	Symptomatic *n* = 18 (72%)	*p* value
Male sex: *n* (%)	15 (60)	5 (71)	10 (56)	0.46
Age at diagnosis (year): median (IQR)	11.1 (2.8–12.8)	11.1 (5.0–12.5)	11.4 (1.3–12.9)	0.88
Age at diagnosis: *n* (%)				**0**.**05**
Age <3 years old	7 (28)	7 (100)	7 (39)	
Age >3 years old	18 (72)	0 (0)	11 (61)	
Status at diagnosis: *n* (%)				**0.05**
Outpatient	11 (44)	6 (86)	5 (28)	
Admitted to ward	11 (44)	1 (14)	10 (56)	
Admitted to ICU	3 (12)	0 (0)	3 (17)	
Pathogenic mutation: *n* (%)	9 (36)	2 (29)	7 (39)	1.00
MYH7	3 (12)	1 (14)	2 (11)	1.00
MYBPC3	2 (8)	1 (14)	1 (6)	0.49
TNN	4 (16)	0 (0)	4 (22)	0.29
Family history of 1st degree relative with cardiomyopathy: *n* (%)	3 (12)	1 (14)	2 (11)	0.83
RCM phenotypes: *n* (%)				
Classic RCM	13 (52)	6 (86)	7 (38)	
RCM with HCM features	7 (28)	0 (0)	7 (39)	**0**.**05**
RCM with LVNC features	5 (20)	1 (14)	4 (22)	0.66

HCM, hypertrophic cardiomyopathy; ICU, intensive care unit; IQR, interquartile range; LVNC, left ventricular non-compaction cardiomyopathy; RCM, restrictive cardiomyopathy.

*p* values equal to or less than 0.05 are bolded.

Symptomatic patients were more likely to have RCM with HCM features than asymptomatic patients (39% vs. 0%, *p* = 0.05). There was no significant difference in incidence of RCM with LVNC features between groups (Table 1). On baseline ECGs, there were no significance differences in terms of presence of left ventricular (LV) hypertrophy (29% vs. 44%, *p* = 0.66), right ventricular (RV) hypertrophy (43% vs. 39%, *p* = 1.00), q-waves (0% vs. 17%, *p* = 0.53), or presence of ischemic changes (14% vs. 44%, *p* = 0.16).

### 3.2. Follow-up care

The median time to primary end-point of death, transplant, or transition to adult services was 0.97 years [IQR: 0.54–2.17]. The asymptomatic group was followed for a longer during the study period than the symptomatic group, (2.26 years [IQR: 0.97–4.11] vs. 0.76 years [IQR: 0.20–1.71] respectively, (*p* = 0.03). On follow-up investigations, there were no significant differences between the asymptomatic and symptomatic groups for the presence of at least moderately reduced LV systolic function (14% vs. 33%, *p* = 0.63), at least moderately reduced RV function (14% vs. 33%, p = 0.63), or at least moderate mitral regurgitation (14% to 17%, *p* = 1.00) on echos (Supplementary Table S1). Of the entire RCM cohort, 40% (*n* = 10) had a cardiac MRI, demonstrating no significant differences between groups for moderate to severely reduced LV systolic function (25% vs. 17%, *p* = 1.00), moderate to severely reduced RV systolic function (25% vs. 0%, *p* = 0.4), or evidence of fibrosis (0% vs. 17%, *p* = 1.00). On follow-up ECGs, there were no significant differences between the asymptomatic and symptomatic groups for the presence of LV hypertrophy (29% vs. 56%, *p* = 0.38), RV hypertrophy (57% vs. 56%, *p* = 0.94), q-waves (0% vs. 22%, *p* = 0.90), ischemic changes (29% vs. 56%, *p* = 0.38), or ventricular ectopy (17% vs. 11%, *p* = 1.00). Similarly, on Holter monitoring, we observed no significant differences in terms of the presence of ischemic changes (43% vs. 56%, *p* = 0.67), ectopy (29% vs. 0%, *p* = 0.47), or ventricular tachycardia (14% vs. 6%, *p* = 0.49) between the 2 groups.

Despite longer follow-up of the asymptomatic group during the study period, we observed no differences between groups in the median number of outpatient cardiac appointments (7 [IQR: 0–16] vs. 4 [IQR: 0–11], *p* = 0.83) or median number of inpatient cardiac admissions (1 [IQR: 0–2] vs. 1 [IQR: 1–3], *p* = 0.2) (Table 2). Additionally, there were no significant differences between groups in exposure to beta-blockers (14% vs. 44%, *p* = 0.35), angiotensin converting enzyme (ACE) inhibitors (0% vs. 11%, *p* = 1.00), or need for inotropic support (43% vs. 28%, *p* = 0.64). Moreover, there were also no significant differences in implantable cardiac defibrillator (ICD) use (29% vs. 22%, *p* = 1.00), extracorporeal membrane oxygenation (ECMO) support (0% vs. 17%, *p* = 0.53), mechanical ventilation (14% vs. 44%, *p* = 0.35), need for CPR (0% vs. 28%, *p* = 0.27), or cardioversion (0% vs. 6%, *p* = 0.52).

**Table 2:** Outcomes of the RCM cohort.

	Total RCM Cohort *n* = 25	Asymptomatic *n* = 7	Symptomatic *n* = 18	*p* value
Age at outcome (years): median (IQR)	13.2 (3.6–15.3)	13.7 (7.4–15.4)	13.0 (2.4–14.8)	0.29
Heart transplant				
Listing: *n* (%)	16 (64)	4 (57)	12 (67)	0.66
Waitlist time (days): median (IQR)	101 (15–273)	98 (9–265)	101 (16–273)	0.57
Heart tx: *n* (%)	12 (48)	4 (57)	8 (44)	0.57
Death: *n* (%)	7 (28)	0 (0)	7 (39)	0.13
Cause of death: *n* (%)				**0.03**
Heart failure	4 (57)	0 (0)	4 (57)	
Sudden cardiac death	2 (29)	0 (0)	2 (29)	
Stroke	1 (14)	0 (0)	1 (14)	
Other cardiac events: *n* (%)				
Inotropes	8 (32)	3 (43)	5 (28)	0.64
ICD	6 (24)	2 (29)	4 (22)	1.00
ECMO	3 (12)	0 (0)	3 (17)	0.53
Mechanical ventilation	9 (36)	1 (14)	8 (44)	0.35
CPR	5 (20)	0 (0)	5 (28)	0.27
Cardioversion	1 (4)	0 (0)	1 (6)	0.52
Adverse cardiac-event composite outcome: *n* (%)	20 (80)	4 (57)	16 (89)	0.11

Adverse cardiac event composite outcome: (CPR, ventilation, cardioversion, ionotropic support, ECMO, tx, death); CPR, cardiopulmonary resuscitation; ECMO, extracorporeal membrane oxygenation; ICD, implantable cardioverter-defibrillator; IQR, interquartile range; RCM, restrictive cardiomyopathy; Tx, transplant.

*p* values equal to or less than 0.05 are bolded.

### 3.3. Transplant-free survival

**Figure 1:**
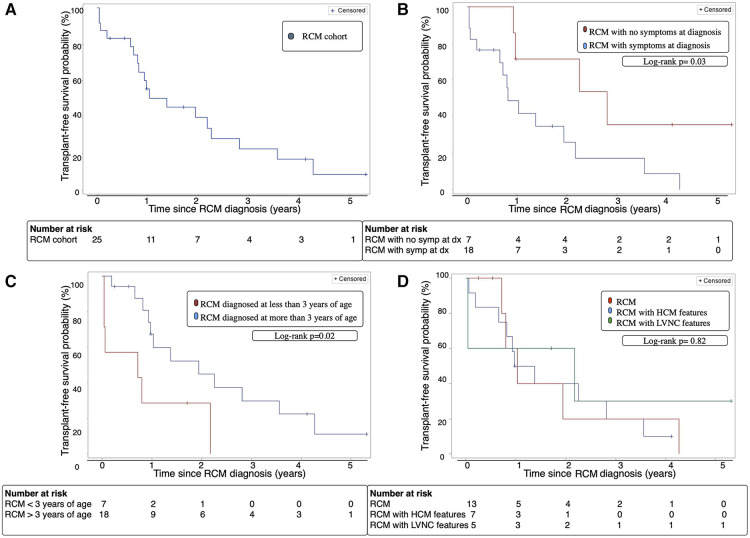
(**A**) Transplant-free survival of the entire RCM cohort over time from diagnosis. (**B**) Transplant-free survival of patients with RCM with and without symptoms at diagnosis over time from diagnosis. (**C**) Transplant-free survival of RCM patients diagnosed before or after age 3 years of age over time from diagnosis. (**D**) Transplant-free survival of cohort with classic RCM phenotype vs. RCM with HCM features vs. RCM with LVNC features. Dx; diagnosis, HCM; hypertrophic cardiomyopathy, LVNC; left ventricular non-compaction cardiomyopathy, RCM; restrictive cardiomyopathy, Symp; symptoms.

At 2 years following RCM diagnosis, the cohort's overall transplant-free survival was 28% [95% CI 16%–61%] (Figure 1A). Amongst the 2 groups, patients without symptoms at diagnosis had a significantly better transplant-free survival 2 years following diagnosis at 57% [95% CI 20%–96%] compared to 17% [95% CI 2%–62%] for patients with symptoms at diagnosis (*p* = 0.03) (Figure 1B). Additionally, patients diagnosed with RCM who were greater than 3 years of age at time of diagnosis had a significantly improved 2-year transplant-free survival compared to patients who were less than 3 years of age [33% vs. 14% (*p* = 0.02)] (Figure 1C). There was no significant difference in 2-year transplant-free survival between the RCM, RCM with HCM features, and RCM with LVNC features [31% vs. 14% vs. 40%, (*p* = 0.82)] (Figure 1D).

Of the entire cohort, 64% patients were listed for heart-transplant during the study period, 4 (57%) patients in the asymptomatic group and 12 (66%) patients in the symptomatic group, *p* = 0.66 (Table 2). At time of listing, half of those patients were listed as high priority (Canadian Pediatric Heart Transplant status 3 and 4) (24). All the asymptomatic patients and 66% symptomatic patients that were listed received a heart transplant during the study period, *p* = 0.57. The median waitlist time was 101 days (15–273), with no significant difference between the groups. There were no deaths in patients asymptomatic at diagnosis during the study period, whereas 39% (*n* = 7) of the symptomatic patients died (*p* = 0.13). The causes of death included heart failure (22%), sudden cardiac death (11%), and stroke (5%).

Independent risk factors for heart-transplant or death included younger age at diagnosis [HR 0.85 (0.76–0.96), *p* = 0.007], symptoms at diagnosis [HR 5.17 (1.28–20.85), *p* = 0.02], and ever requiring admission to the ICU [HR 6.3 (1.77–22.47), *p* = 0.005] (Table 3).

**Table 3:** Risk factors for heart transplant or death in the RCM cohort.

	Univariate analysis			Multivariate analysis		
Risk factors for heart transplant or death	Hazard ratio	95th CI	*p* value	Hazard ratio	95th CI	*p* value
Age at diagnosis	0.90	0.81–0.99	**0**.**04**	0.85	0.76–0.96	**0**.**007**
RCM with HCM features	0.91	0.32–2.6	0.87			
RCM with LVNC features	1.48	0.41–5.21	0.54			
Symptoms at diagnosis	2.93	0.95–9.06	0.06	5.17	1.28–20.85	**0**.**02**
Q wave on baseline ECG	3.18	0.84–12.08	0.09			
Presence of ischemic changes on baseline ECG	3.36	0.95–11.87	0.06			
Ever admitted to ICU	3.04	1.09–8.47	**0**.**03**	6.3	1.77–22.47	**0**.**005**
Ever received mechanical ventilation	3.22	1.18–8.76	**0**.**02**			

ECG, electrocardiogram; HCM, hypertrophic cardiomyopathy; ICU, intensive care unit; LVNC, left ventricular non-compaction cardiomyopathy; RCM, restrictive cardiomyopathy.

*p* values equal to or less than 0.05 are bolded.

### 3.4. Cardiac event-free survival

**Figure 2:**
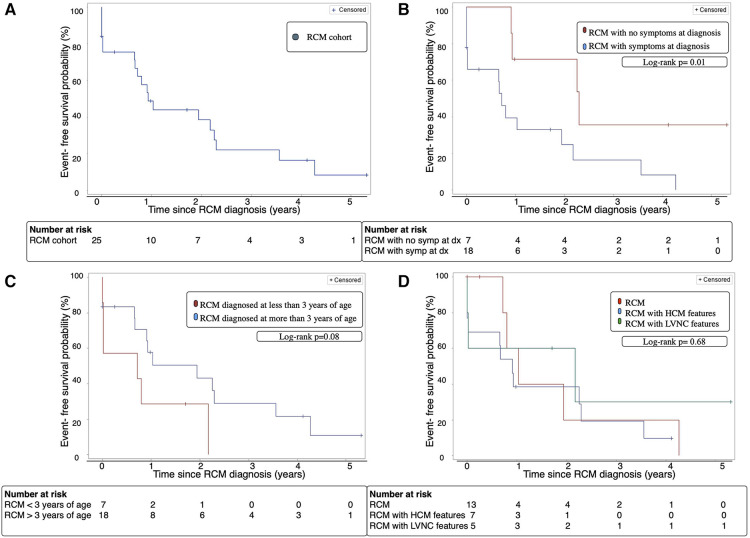
(**A**) Cardiac event-free survival of the entire RCM cohort. (**B**) Cardiac event-free survival of patients with RCM with and without symptoms at diagnosis. (**C**) Cardiac event-free survival of RCM patients diagnosed before or after age 3 years. (**D**) Cardiac event-free survival of cohort with classic RCM phenotype vs. RCM with HCM features vs. RCM with LVNC features. Cardiac event: cardiopulmonary resuscitation, cardioversion, inotropic support, mechanical ventilation, mechanical circulatory support, heart transplant or death, Dx; diagnosis, HCM; hypertrophic cardiomyopathy, LVNC; left ventricular non-compaction cardiomyopathy, RCM; restrictive cardiomyopathy, Symp; symptoms.

As described above, cardiac event-free survival was defined as survival without incurring CPR, cardioversion, inotropic support, mechanical ventilation, MSC, or heart transplant. The overall cardiac event free survival was 39% at 2 years after diagnosis (Figure 2A).

Patients who were asymptomatic at diagnosis had significantly improved cardiac event-free survival after 2 years compared to symptomatic patients; 71% [95% CI 15–93] vs. 25% [95% CI 3–61], *p* = 0.01 (Figure 2B). Event-free survival was not significantly different for younger RCM patients (43% vs. 29%, *p* = 0.08) (Figure 2C), or those with RCM, RCM with HCM features, and RCM with LVNC features (38% vs. 20% vs. 60%, *p* = 0.68) (Figure 2D).

### 3.5. Time to symptom development

**Figure 3:**
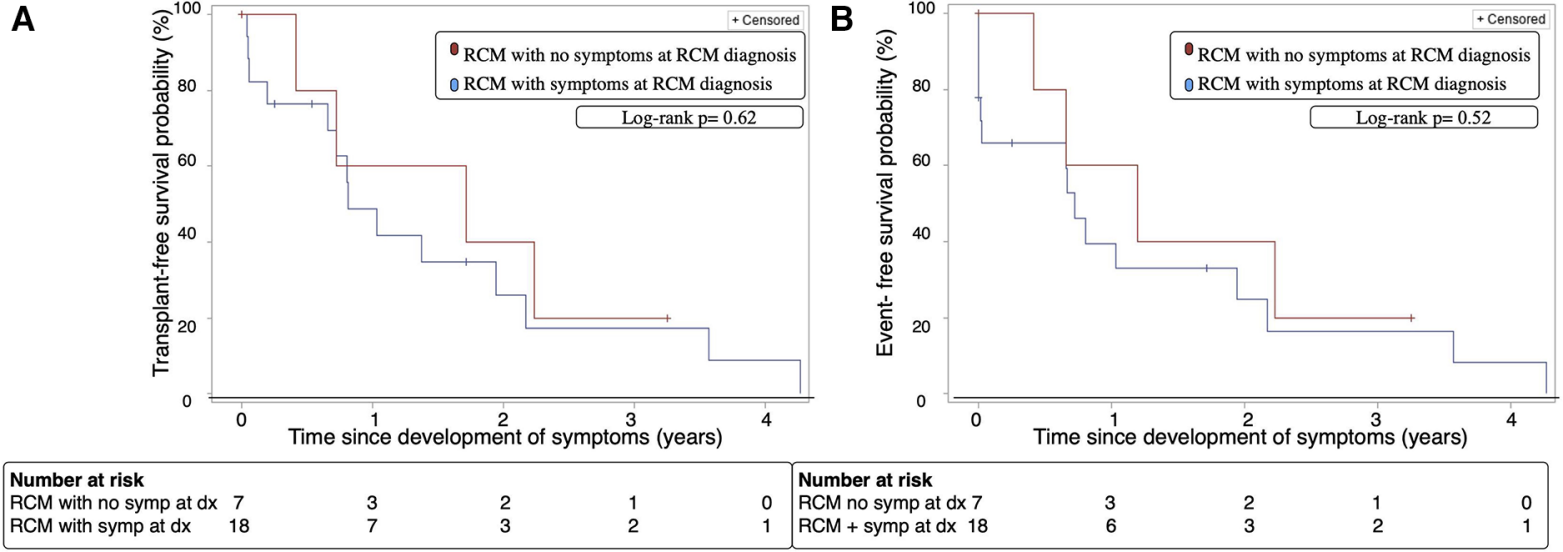
(**A**) Transplant-free survival of patients with RCM with and without symptoms at diagnosis over time from onset of symptoms. (**B**) Cardiac-event free survival of patients with RCM with and without symptoms at diagnosis over time from onset of symptoms. Event; cardiopulmonary resuscitation, cardioversion, inotropic support, mechanical ventilation, mechanical circulatory support, heart transplant or death, Dx; diagnosis, RCM; restrictive cardiomyopathy, Symp; symptoms.

The median time for symptom development in the asymptomatic group was 11.9 months [IQR:3.0–24.7]. When we assessed RCM patients who were initially asymptomatic from the time they developed symptoms, there were no significant differences in transplant-free survival at 2 years after symptom onset (40% vs. 26%, *p* = 0.5, Figure 3A) or cardiac event-free survival at 2 years after symptom onset (40% vs. 25%, *p* = 0.36, Figure 3B) compared to patients who were symptomatic at time of diagnosis.

## 4. Discussion

### 4.1. Symptomatic presentation and outcomes

In this 16-year cohort study, we provide insights into the significance of cardiac symptom development on outcomes of pediatric RCM. We found that patients with any cardiac symptoms at time of diagnosis had a significantly worse transplant-free survival compared to asymptomatic patients. In addition, patients symptomatic at diagnosis were more likely to experience adverse cardiac events including resuscitated cardiac arrest, need for inotropic support, mechanical ventilation, and MCS. Furthermore, repeat time-to-event analysis performed from time of onset of symptoms for those who developed symptoms after initial diagnosis showed similar time to death or transplant from symptom onset as those with symptoms at diagnosis. This emphasizes the importance of prompt evaluation and listing in RCM patients presenting with cardiac symptoms.

Asymptomatic patients in our cohort were followed for longer with overall lower burden of care needs (fewer outpatient visits and admissions per year) than those patients who were symptomatic at time of diagnosis. However, ultimately the proportion of patients requiring advanced heart failure therapies, including MCS and ICDs, was similar between groups, and outcomes after onset of symptoms were similar to those symptomatic at time of diagnosis. Similar findings have been shown in previous studies, with the North American Pediatric Cardiomyopathy Registry (PCMR) group demonstrating that CHF at RCM diagnosis was an independent risk factor for time to death or transplant (2). Wittekind et al. also found that greater than 2 heart failure admissions was associated with a worse composite outcome of death, transplant, MCS, and cardiac arrest (25). While our database did not include detailed information on reason for initial referral, our data suggests that patients presenting for screening with early disease have a less hazardous risk profile during early follow-up than those presenting with cardiac symptoms.

### 4.2. Age at presentation and outcomes

Consistent with previous reports, our study also demonstrated that patients who were younger at diagnosis of RCM were at increased risk of adverse outcomes, with children diagnosed at less than 3 years of age having both a worse transplant-free survival and cardiac-event-free survival (16). On multivariate analysis, younger age at diagnosis remained an independent risk factor for heart transplant or death. While variability exists in previous reports of risk of death vs. transplant in infants with RCM, which may be influenced by transplant listing practices, our data clearly illustrates a poor cardiac risk profile in this cohort with increased need for advanced cardiac therapies (25). The correlation between younger age at diagnosis and poor outcomes is likely multifactorial, contributed to by difficulty assessing and diagnosing heart failure symptoms in infants, challenges with ICD insertion in this cohort, lack of viable options for durable MCS, and poorer outcomes on MCS. This is largely due to challenges achieving adequate decompression using conventional left ventricular apical cannulation in the presence of restrictive physiology with enlarged atria. Atrial cannulation may be considered, but is particularly challenging in smaller infants, and existing data on MCS use in children with RCM suggests significantly lower survival rates on support in those less than 3 years at time of implant (26).

### 4.3. Transplant listing in pediatric RCM

Given survival outcomes and long-term morbidities after transplant, and geographic variations in waitlist times and listing priority for RCM patients, ascertaining the most appropriate time for listing remains a challenge (27–29). This is further complicated by the risk of evolving pulmonary hypertension in those with significant diastolic dysfunction and left atrial hypertension-increasing waitlist and perioperative risks. Compared to other forms of cardiomyopathies, which are characterized by systolic ventricular dysfunction, and where clear guidelines exist on timing of heart transplant listing, listing practices for RCM are largely center dependent due to lack of consensus statements (2, 15–17). Recent changes in the US Organ Procurement and Transplantation Network (OPTN) transplant listing criteria deemed inotrope dependent RCM patients no longer eligible for status 1A, and RCM patients listed under 1 year of age automatically status 1B eligible. However, studies have demonstrated this change to have lead to increased mortality in RCM patients not listed as status 1A exception. The Canadian Heart Transplant Listing Criteria affords a higher listing status (higher priority akin to United Network for Organ Sharing (UNOS) status 1B) to those with RCM over other cardiomyopathies who do not need hospitalization. At our institution, medical management of RCM patients includes discussion around ICD implantation, diuretics for symptom control, and anticoagulation/antiplatelet therapy due to the risk of thromboembolism. ACE-inhibitors and beta-blocker are not routinely used in these patients. The majority of patients that present with RCM undergo a discussion and evaluation for heart transplantation*.* The decision to list a patient for transplantation is dependent on multiple factors, pulmonary vascular resistance being one of them. However, we do not employ a hard cut-off in this group, but rather anticipate the need for aggressive right ventricular support post-transplant, including MCS. Prior to transplantation, the suitable donor organ is selected based on appropriate size match, which needs to be individualized based on recipient size and cardiac size on imaging. Our data, demonstrating variability in outcomes based on clinical status at time of presentation, reiterates the need for larger multicenter cohort studies to facilitate appropriate risk stratification of pediatric RCM patients and better inform transplant listing practices (30–36).

### 4.4. Outcomes in RCM with LVNC or HCM features

A unique aspect of this study was the assessment of myocardial phenotypes in RCM, which includes patients across the spectrum with RCM with HCM features and RCM with LVNC features. When compared to the classic RCM cohort, there were no differences in transplant-free survival or cardiac event-free survival amongst patients with RCM with HCM features and RCM with LVNC features. Our study excluded patients with congenital heart disease and metabolic disease, and evaluated only RCM phenotypes that did not fulfill diagnostic criteria for other types of cardiomyopathies (HCM or LVNC), therefore placing an emphasis on predominantly restrictive disease secondary to RCM. This would suggest that in isolated RCM, the appearance of hypertrophied or trabeculated myocardium does not influence outcomes. However, it has been shown that HCM with restrictive physiology, though still a severe entity, may not have the same survival outcomes as RCM, highlighting the importance of careful attention to functional imaging at the time of diagnosis (2, 37).

### 4.5. Limitations

This was a single center, retrospective study with a small sample size. As such, we were underpowered to study the relationship between individual clinical and genetic parameters and outcomes. Given the long period under study and evolution of echocardiographic protocols for diastolic function over time, we were unable to provide a detailed assessment of diastolic function or analyze the relationship between echocardiographic parameters and outcomes. However, given the rarity of pediatric RCM, many studies to-date have also limited by similar factors (5–12, 25, 38–39).

## 5. Conclusion

Children with RCM remain a high-risk group with a significantly increased risk of death or cardiac events than other cardiomyopathy cohorts. Our data delineates the significant difference in transplant and cardiac event-free survival outcomes in those presenting with cardiac symptoms vs. those presenting asymptomatic or on screening, and indicates the need for urgent evaluation and listing in those presenting at younger age or with cardiac symptoms. While cardiac transplant remains the only viable life-prolonging intervention in RCM, this study highlights the need for further research to facilitate risk stratification and predict optimal timing of transplant listing in patients presenting with early disease.

## Data Availability

The original contributions presented in the study are included in the article/**Supplementary Material**, further inquiries can be directed to the corresponding author.

## References

[1] Price JF Jeewa A Denfield SW. Clinical characteristics and treatment of cardiomyopathies in children. CCR. (2016) 12(2):85–98. 10.2174/1573403X12666160301115543 PMC4861947 26926296

[2] Webber SA Lipshultz SE Sleeper LA Lu M Wilkinson JD Addonizio LJ Outcomes of restrictive cardiomyopathy in childhood and the influence of phenotype: a report from the pediatric cardiomyopathy registry. Circulation. (2012) 126(10):1237–44. 10.1161/CIRCULATIONAHA.112.104638 22843787

[3] Albakri A. Restrictive cardiomyopathy: a review of literature on clinical status and meta-analysis of diagnosis and clinical management. Pediatr Dimens. (2018) 3(2):1–14. 10.15761/PD.1000169

[4] Maron BJ Towbin JA Thiene G Antzelevitch C Corrado D Arnett D Contemporary definitions and classification of the cardiomyopathies. Circulation. (2006) 113(14):1807–16. 10.1161/CIRCULATIONAHA.106.174287 16567565

[5] Weller RJ Weintraub R Addonizio LJ Chrisant MRK Gersony WM Hsu DT. Outcome of idiopathic restrictive cardiomyopathy in children. Am J Cardiol. (2002) 90(5):501–6. 10.1016/S0002-9149(02)02522-5 12208410

[6] Cetta F O’Leary PW Seward JB Driscoll DJ. Idiopathic restrictive cardiomyopathy in childhood: diagnostic features and clinical course. Mayo Clin Proc. (1995) 70(7):634–40. 10.4065/70.7.634 7791385

[7] Anderson HN Cetta F Driscoll DJ Olson TM Ackerman MJ Johnson JN. Idiopathic restrictive cardiomyopathy in children and young adults. Am J Cardiol. (2018) 121(10):1266–70. 10.1016/j.amjcard.2018.01.045 29526277

[8] Chen SC Balfour IC Jureidini S. Clinical spectrum of restrictive cardiomyopathy in children. J Heart Lung Transplant. (2001) 20(1):90–2. 10.1016/S1053-2498(00)00162-5 11166616

[9] Lewis AB. Clinical profile and outcome of restrictive cardiomyopathy in children. Am Heart J. (1992) 123(6):1589–93. 10.1016/0002-8703(92)90814-C 1595540

[10] Russo LM. Idiopathic restrictive cardiomyopathy in children. Heart. (2005) 91(9):1199–202. 10.1136/hrt.2004.043869 16103558 PMC1769097

[11] Hayashi T Tsuda E Kurosaki K Ueda H Yamada O Echigo S. Electrocardiographic and clinical characteristics of idiopathic restrictive cardiomyopathy in children. Circ J. (2007) 71(10):1534–9. 10.1253/circj.71.1534 17895547

[12] Denfield SW Rosenthal G Gajarski RJ Bricker JT Schowengerdt KO Price JK Restrictive cardiomyopathies in childhood. Etiologies and natural history. Tex Heart Inst J. (1997) 24(1):38–44. 9068138 PMC325396

[13] Muchtar E Blauwet LA Gertz MA. Restrictive cardiomyopathy: genetics, pathogenesis, clinical manifestations, diagnosis, and therapy. Circ Res. (2017) 121(7):819–37. 10.1161/CIRCRESAHA.117.310982 28912185

[14] Kucera F Fenton M. Update on restrictive cardiomyopathy. Paediatr Child Health (Oxford). (2017) 27(12):567–71. 10.1016/j.paed.2017.10.002

[15] Dipchand AI Naftel DC Feingold B Spicer R Yung D Kaufman B Outcomes of children with cardiomyopathy listed for transplant: a multi-institutional study. J Heart Lung Transplant. (2009) 28(12):1312–21. 10.1016/j.healun.2009.05.019 19782592

[16] Zangwill SD Naftel D L’Ecuyer T Rosenthal D Robinson B Kirklin JK Outcomes of children with restrictive cardiomyopathy listed for heart transplant: a multi-institutional study. J Heart Lung Transplant. (2009) 28(12):6. 10.1016/j.healun.2009.06.028 19783176

[17] Mehra MR Canter CE Hannan MM Semigran MJ Uber PA Baran DA The 2016 international society for heart lung transplantation listing criteria for heart transplantation: a 10-year update. J Heart Lung Transplant. (2016) 35(1):1–23. 10.1016/j.healun.2015.10.023 26776864

[18] Cardoso B Jeewa A Minn S Ashkanase J Lynch A Jean-St-Michel E. Left ventricular noncompaction cardiomyopathy: left ventricular dilation and dysfunction at baseline portend the risk of death or heart transplantation. Can J Cardiol. (2022) 38(6):754–62. 10.1016/j.cjca.2022.01.030 35122937

[19] Gersh BJ Maron BJ Bonow RO Dearani JA Fifer MA Link MS 2011 ACCF/AHA guideline for the diagnosis and treatment of hypertrophic cardiomyopathy: executive summary: a report of the American college of cardiology foundation/American heart association task force on practice guidelines. Circulation. (2011) 124(24):2761–96. 10.1161/CIR.0b013e318223e230 22068435

[20] Gebhard C Stähli BE Greutmann M Biaggi P Jenni R Tanner FC. Reduced left ventricular compacta thickness: a novel echocardiographic criterion for non-compaction cardiomyopathy. J Am Soc Echocardiogr. (2012) 25(10):1050–7. 10.1016/j.echo.2012.07.003 22883316

[21] Lopez L Colan S Stylianou M Granger S Trachtenberg F Frommelt P Relationship of echocardiographic Z scores adjusted for body surface area to age, sex, race, and ethnicity: the pediatric heart network normal echocardiogram database. Circ Cardiovasc Imaging. (2017) 10(11):e006979. 10.1161/CIRCIMAGING.117.006979 29138232 PMC5812349

[22] Marcus FI McKenna WJ Sherrill D Basso C Bauce B Bluemke DA Diagnosis of arrhythmogenic right ventricular cardiomyopathy/dysplasia: proposed modification of the task force criteria. Eur Heart J. (2010) 31(7):806–14. 10.1093/eurheartj/ehq025 20172912 PMC2848326

[23] Jean-St-Michel E Meza JM Maguire J Coles J McCrindle BW. Survival to stage II with ventricular dysfunction: secondary analysis of the single ventricle reconstruction trial. Pediatr Cardiol. (2018) 39(5):955–66. 10.1007/s00246-018-1845-4 29520465

[24] Isaac D Chan M Haddad H Cheung A. Cardiac Transplantation: Eligibility and Listing Criteria in Canada.:13.

[25] Wittekind SG Ryan TD Gao Z Zafar F Czosek RJ Chin CW Contemporary outcomes of pediatric restrictive cardiomyopathy: a single-center experience. Pediatr Cardiol. (2019) 40(4):694–704. 10.1007/s00246-018-2043-0 30542921

[26] Su JA Menteer J. Outcomes of Berlin heart EXCOR® pediatric ventricular assist device support in patients with restrictive and hypertrophic cardiomyopathy. Pediatr Transplant. (2017) 21(8):1–6. 10.1111/petr.13048 28905470

[27] Singh TP Cherikh WS Hsich E Harhay MO Hayes D Jr Perch M International society for heart and lung transplantation. The international thoracic organ transplant registry of the international society for heart and lung transplantation: twenty-fifth pediatric heart transplantation report-2022; focus on infant heart transplantation. J Heart Lung Transplant. (2022) 41(10):1357–65. 10.1016/j.healun.2022.07.019 35989143 PMC10281815

[28] Rossano JW Cherikh WS Chambers DC Goldfarb S Jr Khush HD KK. The international thoracic organ transplant registry of the international society for heart and lung transplantation: twenty-first pediatric heart transplantation report-2018; focus theme: multiorgan transplantation. J Heart Lung Transplant. (2018) 37(10):1184–95. 10.1016/j.healun.2018.07.018 30293614

[29] Alcorn JB. Changes to OPTN Bylaws and Policies from actions at June Board of Directors Meeting. Available at: https://optn.transplant.hrsa.gov/media/1822/optn_policy_notice_07-24-2014.pdf (Accessed January 10, 2019).

[30] Merlo M Cannatà A Gobbo M Stolfo D Elliott PM Sinagra G. Evolving concepts in dilated cardiomyopathy. Eur J Heart Fail. (2018) 20(2):228–39. 10.1002/ejhf.1103 29271570

[31] van Velzen HG Schinkel AFL Baart SJ Oldenburg RA Frohn-Mulder IME van Slegtenhorst MA Outcomes of contemporary family screening in hypertrophic cardiomyopathy. Circ Genom Precis Med. (2018) 11(4):e001896. 10.1161/CIRCGEN.117.001896 29661763

[32] Ommen SR Mital S Burke MA Day SM Deswal A Elliott P 2020 AHA/ACC guideline for the diagnosis and treatment of patients with hypertrophic cardiomyopathy. Circulation. (2020) 142(25):e558–631. 10.1161/CIR.0000000000000937 33215931

[33] McNally EM Mestroni L. Dilated cardiomyopathy. Circ Res. (2017) 121(7):731–48. 10.1161/CIRCRESAHA.116.309396 28912180 PMC5626020

[34] Parker LE Landstrom AP. The clinical utility of pediatric cardiomyopathy genetic testing: from diagnosis to a precision medicine-based approach to care. Prog Pediatr Cardiol. (2021) 62:101413. 10.1016/j.ppedcard.2021.101413 34776723 PMC8579834

[35] Chintanaphol M Orgil BO Alberson NR Towbin JA Purevjav E. Restrictive cardiomyopathy: from genetics and clinical overview to animal modeling. RCM. (2022) 23(3):108. 10.31083/j.rcm2303108 35345275

[36] Davies RR Haldeman S Pizarro C. Regional variation in survival before and after pediatric heart transplantation–an analysis of the UNOS database. Am J Transplant. (2013) 13(7):1817–29. 10.1111/ajt.12259 23714390

[37] Maskatia SA Decker JA Spinner JA Kim JJ Price JF Jefferies JL Restrictive physiology is associated with poor outcomes in children with hypertrophic cardiomyopathy. Pediatr Cardiol. (2012) 33(1):141–9. 10.1007/s00246-011-0106-6 21892651

[38] Murtuza B Fenton M Burch M Gupta A Muthialu N Elliott MJ Pediatric heart transplantation for congenital and restrictive cardiomyopathy. Ann Thorac Surg. (2013) 95(5):1675–84. 10.1016/j.athoracsur.2013.01.014 23561807

[39] Rivenes SM Kearney DL Smith EO Towbin JA Denfield SW. Sudden Death and Cardiovascular Collapse in Children With Restrictive Cardiomyopathy.:7. 10.1161/01.cir.102.8.876 10952956

