# Supplementary material for: Symptomatic presentation influences outcomes in pediatric restrictive cardiomyopathy
Source: Front Pediatr. 2023 Oct 19;11:1264751. doi: 10.3389/fped.2023.1264751 (PMC10620919; doi:10.3389/fped.2023.1264751)
Supplement: Supplementary file 1 [file Table1.docx]

**Supplementary Table 1.** Investigations during follow-up of the RCM cohort.

|  | **Total RCM Cohort**  **n=25** | **Asymptomatic**  **n=7** | **Symptomatic**  **n=18** | ***p* value** |
| --- | --- | --- | --- | --- |
| Duration of follow-up (yrs): median (IQR) | 0.97 (0.54-2.17) | 2.26 (0.97-4.11) | 0.76 (0.20-1.71) | **0.03** |
| **Echocardiogram** |  | | | |
| Ever documented on any echocardiogram: n (%) |  | | | |
| At least moderately reduced LV function | 7 (28) | 1 (14) | 6 (33) | 0.63 |
| At least moderately reduced RV function | 7 (28) | 1 (14) | 6 (33) | 0.63 |
| At least moderate mitral regurgitation | 4 (16) | 1 (14) | 3 (17) | 1.00 |
| At least moderate tricuspid regurgitation | 4 (16) | 2 (29) | 2 (11) | 0.55 |
| **Cardiac MRI** | 10 (40) | 4 (57) | 6 (33) | 0.28 |
| Ever documented on any cardiac MRI: n (%) |  | | | |
| Moderate to severely reduced LV function | 2 (20) | 1 (25) | 1 (17) | 1.00 |
| Moderate to severely reduced RV function | 1 (10) | 1 (25) | 0 (0) | 0.40 |
| Presence of fibrosis | 1 (10) | 0 (0) | 1 (17) | 1.00 |
| Presence of gadolinium | 1 (10) | 0 (0) | 1 (17) | 1.00 |
| **ECG** | 25 (100) | 7 (100) | 18 (100) |  |
| Ever documented on any ECG: n (%) |  | | | |
| Left ventricular hypertrophy | 12 (48) | 2 (29) | 10 (56) | 0.38 |
| Right ventricular hypertrophy | 14 (56) | 4 (57) | 10 (56) | 0.94 |
| Q wave | 4 (16) | 0 (0) | 4 (22) | 0.90 |
| Ischemic changes | 12 (48) | 2 (29) | 10 (56) | 0.38 |
| Ventricular ectopy | 3 (12) | 1 (17) | 2 (11) | 1.00 |
| **Holter** | 25 (100) | 7 (100) | 18 (100) | 0.18 |
| Ever documented on any holter: n (%) |  | | | |
| Ischemic changes | 13 (52) | 3 (43) | 10 (56) | 0.67 |
| Ventricular ectopy | 2 (8) | 2 (29) | 0 (0) | 0.47 |
| Ventricular tachycardia | 2 (8) | 1 (14) | 1 (6) | 0.49 |

ECG electrocardiogram; IQR interquartile range; LV left ventricle; MRI magnetic resonance imaging; RCM restrictive cardiomyopathy; RV right ventricle; Yr year.

*p* values equal to or less than 0.05 are bolded.
